# Effects of First Time Minimum Volume Regulation on the Surgical Treatment of Lung Cancer in Germany

**DOI:** 10.3390/cancers18121878

**Published:** 2026-06-09

**Authors:** Julia Riedel, Michael Ried, Erich Hecker, Stephan Eggeling, Martin Utzig, Hans-Stefan Hofmann

**Affiliations:** 1 Department of Thoracic Surgery, University Hospital Regensburg, 93053 Regensburg, Germany; michael.ried@ukr.de (M.R.); hans-stefan.hofmann@ukr.de (H.-S.H.); 2 Thoracic Center Ruhrgebiet, Department of Thoracic Surgery, Evangelisches Hospital Herne, 44623 Herne, Germany; e.hecker@evk-herne.de; 3 Department of Thoracic Surgery, Vivantes Hospital Neukölln, 12351 Berlin, Germany; stephan.eggeling@vivantes.de; 4 German Cancer Society, 14057 Berlin, Germany; utzig@krebsgesellschaft.de; 5 Department of Thoracic Surgery, Hospital Barmherzige Brüder Regensburg, 93049 Regensburg, Germany

**Keywords:** lung carcinoma, minimum volume regulation, lung cancer center, thoracic surgical care

## Abstract

**Objectives:** In 2021, the German Federal Joint Committee (G-BA) introduced minimum volume regulations (MVRs) for the surgical treatment of lung cancer with the aim of improving quality of care through centralization. The present study aimed to evaluate the impact of this regulation on the development of lung cancer surgery in Germany. **Methods:** We performed a retrospective analysis based on the AOK (medical insurance) minimum volume transparency lists (2024–2026) and certification data from the German Cancer Society (DKG). Primary endpoint was the development of hospital locations according to MVRs. Secondary endpoints included the number of operations, the number of DKG-certified lung cancer centers (LCs), and the percentage of procedures performed in DKG-certified LCs. **Results:** Nationwide, the number of hospital locations fell by 19.6% between January 2022 and June 2025 following the implementation of the MVRs, while the number of anatomical lung resections increased by 11.3%. The proportion of procedures performed at hospital locations with ≥75 operations per year (≙MVRs) increased from 42.3% to 82.2%. The number of DKG-certified LCs went up by 24.2% (62 to 77), and the percentage of resections performed there went from 56.6% to 69%. Lastly, 17.8% of hospital sites still got a billing permission, even though they did not meet the MVRs. **Conclusions:** Implementation of the MVRs was associated with substantial structural centralization of lung cancer surgery in Germany, characterized by fewer hospital sites (−19.6%), increased surgical volumes (+11.3%), and a growing proportion of care delivered in DKG-certified LCs (+24.2%).

## Simple Summary

This study explores how the introduction of a minimum volume regulation for lung cancer surgery in Germany has influenced the organization of healthcare delivery. The regulation aims to improve quality of care by concentrating complex procedures in more experienced hospitals. Using nationwide data, we assessed how care structures have changed since its implementation. We found that fewer hospitals now perform lung cancer surgery, while the overall number of procedures has increased. At the same time, a growing proportion of patients are treated in specialized lung cancer centers. These changes indicate a clear shift toward more centralized care, which may contribute to improved treatment outcomes. Our findings provide valuable insights for clinicians, researchers, and policymakers on how such regulations can shape healthcare systems and the delivery of cancer care.

## 1. Introduction

Lung cancer is one of the most common malignant diseases in Germany and is still associated with a high mortality rate [1]. Against this background, optimizing the quality of care is of considerable importance in health policy. A key instrument for quality assurance is the specification of minimum volumes (MVs). As early as 2007, Kaiser called for MVs for thoracic surgery in order to be able to provide adequate complication management through the corresponding practice and experience effect, with the aim of achieving lower surgery-related mortality [2].

International experience supports centralization strategies. Data from Denmark demonstrated that lung cancer surgery concentrated in four university-based high-volume centers is associated with high minimally invasive resection rates, low perioperative mortality, and improved long-term survival [3]. Similarly, European structural analyses by the EACTS–ESTS working group have emphasized the importance of specialized thoracic units and defined service volumes to ensure quality and training standards [4,5].

Beyond international evidence, these findings are consistently replicated in national healthcare data showing a consistent inverse association between hospital volume and in-hospital mortality across a broad range of inpatient treatments, particularly in complex surgical procedures [6].

Evidence supporting the volume–outcome relationship in surgery is well established. Higher hospital and surgeon procedure volumes have been associated with lower operative mortality across multiple surgical specialties, including oncologic surgery, such as lung cancer surgery, with improved postoperative and long-term outcomes after pulmonary resection observed in high-volume centers [7,8,9].

The highest decision-making body in the German health system—the Joint Federal Committee (G-BA)—sets minimum volume requirements (MVRs) for selected, plannable hospital services since the early 2000s [10,11]. On 16 December 2021, MVs were set for the first time for the surgical treatment of lung cancer. After a transition phase in 2024 with an MV of ≥40 anatomical lung resections per year and location, there was a nationwide binding annual MV of ≥75 anatomical lung resections for lung cancer or lung metastases per hospital location [12,13]. This requirement also corresponds to the case number criteria for primary surgical cases (=anatomical lung resection for lung cancer) and expert cases (=anatomical lung resection for lung metastases) within the framework of the certification of lung cancer centers (LCs) by the German Cancer Society (DKG) [14]. The certification system was developed as a structured quality assurance approach with defined minimum structural and process requirements aimed at standardizing oncological care across hospitals [15].

Recent evidence from Germany suggests that certification of specialized oncologic centers may positively influence patient outcomes, with large-cohort studies demonstrating significant survival advantages from patients treated in DKG-certified breast, pancreatic, and head and neck cancer hospitals compared with non-certified hospitals [16,17,18,19].

In 2019, surgical treatment for lung cancer was performed at 328 of the 1.914 hospital locations in Germany. With the implementation of the MVRs in the surgical treatment of lung cancer, the G-BA predicted a reduction in the number of locations in Germany to around 90 clinics, thereby achieving an improvement in the quality of treatment [20]. The aim of this study was to determine the extent to which this prediction has been realized and to show its consequences on the thoracic surgery landscape in Germany. At the same time, the developments and effects of the MVRs on DKG-certified LCs were also investigated. In order to highlight regional developments and differences in care, a separate analysis of the MVRs was also carried out in three selected federal states.

## 2. Materials and Methods

The AOK minimum volume transparency list provides nationwide online data that can be used to analyze hospital locations and operation numbers. The transparency lists for 2024, 2025, and 2026 were evaluated. These lists cover all hospital locations in Germany that have been performing minimum volume-relevant surgeries since 2020 and are authorized to bill health insurance companies [21,22,23]. The number of operations was available for the included hospital locations for the period from January 2022 to June 2025. The number of cases at the hospital locations is given in annual intervals. For the evaluation, a division was made into the phase before the MVRs (July 2022–June 2023), the transition phase (July 2023–June 2024), and the implementation phase (July 2024–June 2025). Only hospitals with a complete dataset were included in the analysis; first-time or repeat-service providers with incomplete operation figures or deviating survey periods were not taken into account. A total of eight hospital locations nationwide were excluded, including three DKG-certified LCs.

The study hypothesis was that the introduction of the MVRs would result in a reduction in the number of hospital locations, an increase in the number of surgical cases, and an increase in the number of patients treated at DKG-certified LCs.

The primary endpoint was the development of the number of hospital locations with billing authorization vis-à-vis health insurance companies. Secondary endpoints included the development of the number of surgeries, the number of DKG-certified LCs, and the proportion of oncological surgeries performed at DKG-certified LCs in accordance with the MVRs.

A distinction was also made between DKG-certified and non-certified hospitals based on the certification data provided by the DKG (start, end, and suspension of certificate validity). Linking the AOK data with the certification data allowed for a differentiated view of DKG-certified and non-certified hospitals.

In addition to a nationwide evaluation, a separate analysis was also carried out for the federal states of Bavaria, Berlin, and North Rhine-Westphalia. These three federal states were selected because they differ significantly in terms of population density, hospital landscape, and historical care structure: Bavaria is a large state, Berlin is the capital and city state, and North Rhine-Westphalia is a highly populated federal state. In addition, the North Rhine-Westphalia Ministry of Labor, Health, and Social Affairs published the North Rhine-Westphalia Hospital Plan 2022 in April 2022, assigning general and specific service groups, and it was implemented in autumn 2022 [24,25].

## 3. Results

### 3.1. Development of Thoracic Surgery Hospital Locations/DKG-Certified LCs Under the MVRs

**Figure 1:**
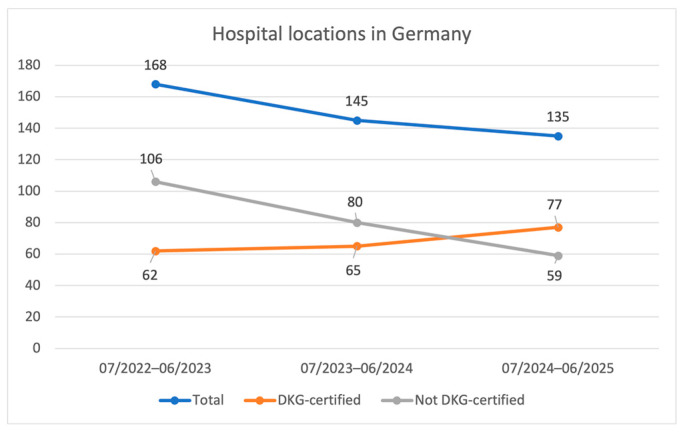
** ** Development of hospital locations performing anatomical lung resections for lung cancer and lung metastases in Germany.

Nationwide, there was a significant drop in the number of hospital locations with positive billing permission from 168 to 135 (−19.6%) locations during the survey period (Table 1, Figure 1). At the start of the MVR transition phase (July 2022–June 2023 to July 2023–June 2024), the number of hospital locations fell by 23 (−13.7%), and in the following period (July 2023–June 2024 to July 2024–June 2025), by a further 10 locations (−6.9%). At the time before the MVs were introduced (July 2022–June 2023), the number of hospitals (*n* = 97) that did not reach the MV of ≥75 operations predominated. During this period, only 71 hospitals (42.3%) achieved the MV of ≥75. Two years later (July 2024–June 2025), the proportion of hospitals that met the MV clearly predominated (*n* = 111). At the same time, 24 hospital locations (17.8%) continued to receive billing permission even though they fell short of the MV.

With the MV being achieved, the number of DKG-certified LCs also increased from 62 to 77 (+24.2%) since the introduction of the MVRs (Table 2). This means that in June 2025, 69% (77/111) of hospitals that met the MVRs were also certificated by the DKG as LCs.

### 3.2. Development of Thoracic Surgery Operation Numbers Under the MVRs

**Figure 2:**
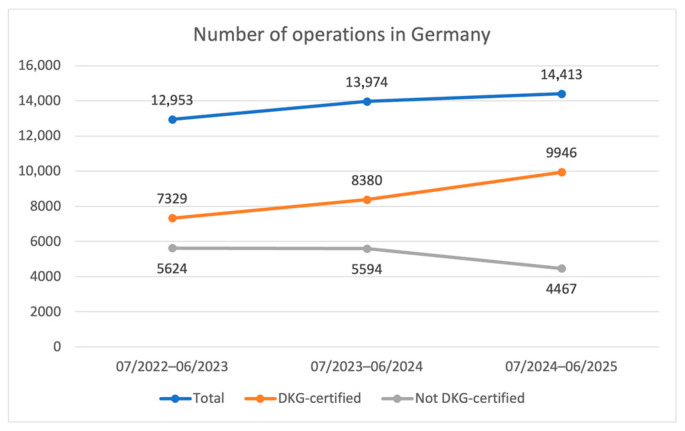
** ** Development in anatomical lung resections for lung cancer and lung metastases in Germany.

The number of anatomical lung resections in accordance with the MVRs increased significantly from 12.953 to 14.413 (+11.3%). Even before the MVRs were introduced, most patients (66.2%) underwent thoracic surgery in hospitals that fulfilled these regulations. This proportion rose to 90.3% at the end of the period under review (July 2024–June 2025). With the increase in DKG-certified LCs, the number of patients who underwent surgery at a DKG-certified LC also climbed from 7.328 at the beginning of the survey period to 9.946 (+35%) at the end (Table 2 and Figure 2). This means that the proportion of patients who underwent surgery at a DKG-certified LC was 69% of all surgeries performed in Germany.

### 3.3. Regional Development Under MVRs

#### 3.3.1. Bavaria

In the state of Bavaria, the number of hospital locations with billing authorization for health insurance companies decreased from 25 to 20 (−20%) between July 2022 and June 2025 (Table 3). The number of procedures increased significantly during the same period, from 1.626 to 2.042 (+24.6%) operations. In addition, the proportion of operations at locations with ≥75 operations/year increased from 66.5% (July 2022–June 2023) to 94.6% (July 2024–June 2025), which was above the German average of 90%. Two hospital locations had recently continued to hold a license to provide services even though they did not meet the MVRs. Neither hospital location ever reached the currently required MV during the entire survey period, but both recorded an increase in the number of operations over the course of the last few years. The proportion of DKG-certified LCs at Bavarian hospital locations that achieved an MV of ≥75 was most recently 55% (10/18), which is below the national average of 69%. In Bavarian DKG-certified LCs, 1.252 of a total of 2.042 operations (61.3%) were performed. This proportion is also below the national average, where 69% of patients were operated on at DKG-certified LCs.

#### 3.3.2. North Rhine-Westphalia (NRW)

In NRW, the number of hospital locations with billing authorization fell to 34 locations (−22.7%; Table 4) during the survey period, slightly higher than the nationwide trend. Seven locations (21%) continued to receive approval to provide services despite not reaching the MV, including a DKG-certified LC, which suspended and reintroduced its certificate several times during this period; as of the last reporting date of 30 June 2025, the certificate had been suspended. The total number of operations in accordance with the MVRs increased in NRW from 3.570 to 3.881 operations (+8.7%). Of these, 68.5% (July 2022 to June 2023) and then 89.2% (July 2024 to June 2025) of the operations were performed at hospital locations with ≥75 operations/year. The number of DKG-certified LCs grew from 16 in 2022 to 19 in 2023 and 23 LCs by June 2025. This means that 85% of hospital locations with MV of ≥75 operations in NRW had a DKG certification as an LC (Germany-wide: only 69%). The currently DKG-certified LCs performed 3.052 of all MV-relevant lung resections in NRW (*n* = 3.881). At 78.6%, this ratio was significantly above the average in Germany (69%).

#### 3.3.3. Berlin

In the German capital, the number of hospital locations performing lung resections relevant to MVRs decreased by one location during the survey period from July 2022 to June 2025 (Table 5). This location, which no longer performs any MV-relevant operations as of 2023, was part of a multi-location LC, where all operations will now be performed at the main location. Berlin is the only federal state in which all hospital locations comply with the MVRs and are also certified as LCs according to DKG criteria. The total number of operations in Berlin increased from 767 to 803 MV operations (+4.7%) during the survey period. Between July 2022 and June 2023, 96.5% of procedures were already performed at hospital locations with ≥75 operations/year. Most recently (July 2024 to June 2025), 100% of operations were performed in DKG-certified LCs.

## 4. Discussion

Analysis of the AOK minimum volume transparency list confirmed that the first nationwide introduction of MVRs for the surgical treatment of malignant lung tumors has been accompanied by a significant structural change in thoracic surgery care in Germany. Of the 328 hospitals that performed anatomical lung resections in 2019, only 135 hospital locations were sill recorded by the AOK in 2025, corresponding to a reduction of almost 60%. The reduction in locations can be interpreted as an indication that previously existing parallel structures with low case numbers have been replaced in favor of larger centers.

Most of the remaining hospitals now meet the required minimum volume targets, corresponding to 111 clinics (82.2%). Before the introduction of the MVRs, this proportion was less than half, at only 42.3%. Although the G-BA originally projected a reduction to approximately 90 clinics, current developments suggest that the number of hospitals fulfilling the MVRs may stabilize at a somewhat higher level due to institutional restructuring and interhospital cooperation. Similar centralization processes after implementation of MV policies have also been reported in other European healthcare systems, particularly in Scandinavian countries with highly centralized thoracic surgical care [3]. However, the centralization observed cannot be attributed solely to the introduction of minimum case numbers. Long-term trends towards increasing specialization in thoracic oncology, demographic changes, and growing financial pressure on smaller hospitals may also have contributed significantly to the structural developments observed.

Currently, the proportion of hospital locations that have been granted approval to provide services despite repeatedly failing to meet the MVRs is 17.8% nationwide. In the short term, such exemptions may be considered necessary by political bodies, particularly in rural regions, but they may also attenuate the intended centralization effect of the MVRs.

Despite the reduction in the number of hospitals, the number of anatomical lung resections subject to the MVRs increased by 11.3% between July 2022 and June 2025. It is pleasing to note that even before the introduction of MV, 2/3 of resections were performed in hospitals with a higher surgical volume (≥75 operations per year). By the end of the period under review (June 2025), this proportion has even increased to 90.3%. Parallel to this development, the number of DKG-certified LCs increased by 24.2%, and 69% of patients were operated on in certified centers in June 2025. The care rate for all patients newly diagnosed with lung cancer in a DKG-certified LC (so-called primary cases) varies significantly from region to region and currently stands at approximately 45.1% nationwide [1,26,27].

The introduction of the MVRs has had varying effects in the federal states under review. While Bavaria and North Rhine-Westphalia showed reductions in hospital locations of approximately 20% comparable to the national trend, thoracic surgical care in Berlin already underwent substantial centralization in the mid-1990s due the state bed plan after reunification. Consequently, all lung cancer resections in Berlin were already performed in hospitals exceeding the MV threshold.

At the state level, Berlin’s already highly centralized healthcare structure was associated with only a moderate increase in surgical volumes (4.7%). In contrast, Bavaria and North Rhine-Westphalia showed more pronounced increases of 24.6% and 8.7%, respectively, alongside reductions in hospital locations of 20% and 22.7%. These findings suggest that the implementation and structural impact of the MVRs are strongly influenced by regional healthcare infrastructure, hospital density, and pre-existing levels of centralization.

The primary objective of this study was not to evaluate clinical outcomes but rather to assess structural changes in thoracic surgical care following implementation of the MVRs. Consequently, the observed concentration of care cannot be directly interpreted as evidence of improved patient outcomes. Nevertheless, the rationale underlying MVR policies is supported by national studies on cancer surgery demonstrating that surgical mortality is significantly lower in hospitals with higher case volumes, which supports the validity of minimum case volumes as a quality indicator [6,7,8,9,28].

This has always been one of the main points of criticism of the MVRs. Currently, only optional DKG certification as an LC is associated with structural and process requirements. The DKG’s certified organ centers also collect key figures that allow for conclusions to be drawn about the quality of the centers. For example, the median 30-day mortality rate for primary cases in DKG-certified LCs in the last few years was between 1.2% (2023) and 1.7% (2021), which is not only below the overall mortality rate for all anatomical lung resections performed in Germany in 2024 for lung cancer (2.4%) but also below that of all facilities with ≥75 anatomical resections (2.1%) [26,29]. In addition, recent German cohort studies, including analyses from the WiZen study, demonstrated associations between treatment in certified organ cancer centers and improved survival outcome in several oncologic entities [16,17,18,19,30,31]. These findings are consistent with a systematic review indicating a trend toward improved care quality in certified oncological centers [32]. On a positive note, the number of DKG-certified LCs has also increased in years past with the introduction of the MVRs.

In the coming years, German hospitals will introduce legally prescribed medical service groups that are subject to binding structural and procedural requirements. This will increasingly implement in Germany the European guidelines on the structure and qualification of general thoracic surgery, as introduced years ago by Kleptko and Brunelli et al. [4,5].

## 5. Conclusions

The introduction of a minimum number of anatomical resections for lung cancer led to a concentration of thoracic surgery centers in Germany. With consistent application of the MVRs, this centralization will continue, with current trend analysis indicating that approximately 120–130 hospital locations can be expected throughout Germany. The analysis of the three German states of Bavaria, North Rhine-Westphalia, and Berlin revealed significant regional differences in the extent and dynamics of the centralization, which are partly due to historical factors but primarily to population density. With regard to the thoracic surgical structures in Europe, this demonstrates that the introduction of MVRs for oncological surgeries is sensible, but that their implementation can vary regionally. Besides the centralization of thoracic surgery units, standards for structural and outcome quality are absolutely essential. With the future introduction of performance groups, this goal will also be implemented as a mandatory requirement in Germany.

### Limitations

The evaluation was based on structural data from the AOK minimum volume transparency lists and certification information from the DKG. Consequently, no patient-level clinical data were available and no conclusions can be drawn regarding clinical endpoints such as morbidity, mortality, or long-term survival. Furthermore, regional differences in tumor characteristics and case complexity could not be taken into account, and it was not possible to adjust for risk. Therefore, the observed structural changes cannot be directly interpreted as evidence of improved patient outcomes. In addition, the observational design carries a potential risk of ecological bias. The available datasets did not include information on tumor board decision-making, thoracic surgical workforce, or departmental resources such as operating theater capacity or bed allocation, limiting assessment of organizational effects at the institutional level.

Nevertheless, the available nationwide data allowed for a robust structural evaluation of the effects of the MVRs on hospital site distribution and surgical case volume development in Germany. Future studies including patient-level outcome data are needed to determine whether the observed centralization translates into measurable clinical benefits.

## Figures and Tables

**Table 1:** Hospital locations and minimum volume-relevant surgery data for lung cancer in Germany [21,22,23].

Germany		Phase Before the MVRs July 2022– June 2023	Δ (*n*; %)	Transitional Phase of the MVRs July 2023– June 2024	Δ (*n*; %)	Implementation Phase of the MVRs July 2024– June 2025
Hospital locations	Total (*n*)	168	−23;	145	−10;	135
			−13.7%		−6.9%	
	≥75 operations (*n*; %)	71;	20;	97;	14;	111;
		42.3%	+28.2%	66.9%	+14.4%	82.2%
	<75 operations (*n*; %)	97;	−49;	48;	−24;	24;
		57.7%	−50.5%	33.1%	−50%	17.8%
Operations	Total (*n*)	12.953	1.021;	13.974	439	14.413
			+7.9%		+3.1%	
	Hospital locations ≥ 75 operations (*n*; %)	8.577;	2861;	11.438;	1.571;	13.009;
		66.2%	+33.4%	81.9%	+13.7%	90.3%
	Hospital locations < 75 operations (*n*; %)	4.376;	−1.807;	2.509;	−1.105;	1.404;
		33.8%	−41.2%	17.9%	−44%	9.7%

**Table 2:** Hospital locations and minimum volume-relevant surgery figures for lung cancer at DKG-certified locations [21,22,23].

DKG-Certified Locations		Phase Before the MVRs July 2022– June 2023	Δ (*n*; %)	Transitional Phase of the MVRs July 2023– June 2024	Δ (*n*; %)	Implementation Phase of the MVRs July 2024– June 2025
**DKG-certified** **hospital locations with active** **certification**	**Total (** ***n*** **)**	62 ^1^	3;	65 ^2^	12;	77 ^3^
			+4.8%		+18.5%	
	**Bavaria (** ***n*** **)**	8	±0	8	2;	10
					25%	
	**NRW (** ***n*** **)**	16	3;	19	4;	23
			+18.8%		+21.1%	
	**Berlin (** ***n*** **)**	6	−1;	5	±0	5
			−16.7%			
**Operations at** **DKG-certified** **hospital locations with active** **certification**	**Total (** ***n*** **)**	7.328	1.052;	8.380	1.566;	9.946
			+14.4%		+18.7%	
	**Bavaria (** ***n*** **)**	894	103;	997	255;	1.252
			+11.5%		+25.6%	
	**NRW (** ***n*** **)**	1.921	431;	2.352	700;	3.052
			+22.4%		+29.8%	
	**Berlin (** ***n*** **)**	767	63;	830	−27;	803
			+8.2%		−3.2%	

^1^: Reference date: 1 July 2022; ^2^: reference date: 1 July 2023; ^3^: reference date: 30 June 2025.

**Table 3:** Hospital locations and minimum volume-relevant surgery data for lung cancer in Bavaria [21,22,23].

Bavaria		Phase Before the MVRs July 2022– June 2023	Δ (*n*; %)	Transitional Phase of the MVRs July 2023– June 2024	Δ (*n*; %)	Implementation Phase of the MVRs July 2024– June 2025
Hospital locations	Total (*n*)	25	−4;	21	−1;	20
			−16%		−4.8%	
	≥75 operations (*n*; %)	10;	3;	13;	5;	18;
		40%	+30%	61.9%	+38.5%	90%
	<75 operations (*n*; %)	15;	−7;	8;	−6;	2;
		60%	−46.7%	38.1%	−75%	10%
Operations	Total (*n*)	1.626	236;	1.862	180;	2.042
			+14%		+9.7%	
	Hospital locations ≥ 75 operations (*n*; %)	1.081;	343;	1.424;	508;	1.932;
		66.5%	+31.7%	76.5%	+35.7%	94.6%
	Hospital locations < 75 operations (*n*; %)	545;	−107;	438;	−328;	110;
		33.5%	−19.6%	23.5%	−74.9%	5.4%

**Table 4:** Hospital locations and minimum volume-relevant surgery data for lung cancer in North Rhine-Westphalia [21,22,23].

North Rhine-Westphalia (NRW)		Phase Before the MVRs July 2022– June 2023	Δ (*n*; %)	Transitional Phase of the MVRs July 2023– June 2024	Δ (*n*; %)	Implementation Phase of the MVRs July 2024– June 2025
Hospital locations	Total (*n*)	44	−7;	37	−3;	34
			−15.9%		−8.1%	
	≥75 operations (*n*; %)	20;	8;	28;	−1;	27;
		45.5%	+40%	75.7%	−3.6%	79.4%
	<75 operations (*n*; %)	24;	−15;	9;	−2;	7;
		54.5%	−62.5%	24.3%	−22.2%	20.6%
Operations	Total (*n*)	3.570	370;	3.940	−59;	3.881
			+10.3%		−1.5%	
	Hospital locations ≥ 75 operations (*n*; %)	2.444;	963;	3.407	53;	3.460;
		68.5%	+39.4%	86.5%	+1.6%	89.2%
	Hospital locations < 75 operations (*n*;%)	1.126;	−593;	533;	−112;	421;
		31.5%	−52.7%	13.5%	−21%	10.8%

**Table 5:** Hospital locations and minimum volume-relevant surgery data for lung cancer in Berlin [21,22,23].

Berlin		Phase Before the MVRs July 2022– June 2023	Δ (*n*; %)	Transitional Phase of the MVRs July 2023– June 2024	Δ (*n*; %)	Implementation Phase of the MVRs July 2024– June 2025
Hospital locations	Total (*n*)	6	−1;	5	±0	5
			−16.7%			
	≥75 operations (*n*; %)	5;	±0	5;	±0	5;
		83.3%		100%		100%
	<75 operations (*n*; %)	1;	−1;	0	±0	0
		16.7%	−100%			
Operations	Total (*n*)	767	63;	830	−27;	803
			+8.2%		−3.2%	
	Hospital locations ≥ 75 operations (*n*; %)	740;	90;	830;	−27;	803;
		96.5%	+19.1%	100%	−3.3%	100%
	Hospital locations < 75 operations (*n*; %)	27;	−27;	0	±0	0
		3.5%	−100%			

## Data Availability

The number of operations and hospital locations was derived from the AOK’s minimum volume transparency lists in the public domain: https://www.aok.de/pp/hintergrund/mindestmengen/mindestmengen-transparenzkarte-2026/ (accessed on 21 November 2025). Information on certification status will be shared on request to the corresponding author with permission of the German Cancer Society (DKG).

## Abbreviations

The following abbreviations are used in this manuscript:

DKG	German Cancer Society, Deutsche Krebsgesellschaft
LC	Lung cancer center
MV	Minimum volume
MVRs	Minimum volume regulations
